# Increasing striatal dopamine release through repeated bouts of theta burst transcranial magnetic stimulation of the left dorsolateral prefrontal cortex. A 18F-desmethoxyfallypride positron emission tomography study

**DOI:** 10.3389/fnins.2023.1295151

**Published:** 2024-01-18

**Authors:** Usman Jawed Shaikh, Antonello Pellicano, Andre Schüppen, Alexander Heinzel, Oliver H. Winz, Hans Herzog, Felix M. Mottaghy, Ferdinand Binkofski

**Affiliations:** ^1^Section Clinical Cognitive Sciences, Department of Neurology, Faculty of Medicine, RWTH Aachen University, Aachen, Germany; ^2^Department of Educational Sciences, University of Catania, Catania, Italy; ^3^Interdisciplinary Center for Clinical Research – Brain Imaging Facility, University Hospital Aachen, Aachen, Germany; ^4^Department of Nuclear Medicine, Faculty of Medicine, RWTH Aachen University, Aachen, Germany; ^5^Research Centre Juelich, Institute of Neuroscience and Medicine (INM-4), Juelich, Germany; ^6^Department of Radiology and Nuclear Medicine, Maastricht University Medical Center (MUMC+), Maastricht, Netherlands; ^7^Juelich Aachen Research Alliance (JARA)—BRAIN, Juelich, Germany

**Keywords:** positron emission tomography (PET), transcranial magnetic stimulation (TMS), intermittent theta burst stimulation (iTBS), prefrontal cortex (PFC), dorso-lateral prefrontal cortex (DLPFC), resting motor threshold (rMT), ANOVA, repeated-measure analysis of variance

## Abstract

**Introduction:**

Transcranial Magnetic Stimulation (TMS) can modulate fronto-striatal connectivity in the human brain. Here Positron Emission Tomography (PET) and neuro-navigated TMS were combined to investigate the dynamics of the fronto-striatal connectivity in the human brain. Employing 18F-DesmethoxyFallypride (DMFP) – a Dopamine receptor-antagonist – the release of endogenous dopamine in the striatum in response to time-spaced repeated bouts of excitatory, intermittent theta burst stimulation (iTBS) of the Left-Dorsolateral Prefrontal Cortex (L-DLPFC) was measured.

**Methods:**

23 healthy participants underwent two PET sessions, each one with four blocks of iTBS separated by 30 minutes: sham (control) and verum (90% of individual resting motor threshold). Receptor Binding Ratios were collected for sham and verum sessions across 37 time frames (about 130 minutes) in striatal sub-regions (Caudate nucleus and Putamen).

**Results:**

Verum iTBS increased the dopamine release in striatal sub-regions, relative to sham iTBS. Dopamine levels in the verum session increased progressively across the time frames until frame number 28 (approximately 85 minutes after the start of the session and after three iTBS bouts) and then essentially remained unchanged until the end of the session.

**Conclusion:**

Results suggest that the short-timed iTBS protocol performed in time-spaced blocks can effectively induce a dynamic dose dependent increase in dopaminergic fronto-striatal connectivity. This scheme could provide an alternative to unpleasant and distressing, long stimulation protocols in experimental and therapeutic settings. Specifically, it was demonstrated that three repeated bouts of iTBS, spaced by short intervals, achieve larger effects than one single stimulation. This finding has implications for the planning of therapeutic interventions, for example, treatment of major depression.

## 1 Introduction

The fronto-striatal networks share a well-established association between the frontal cortex and sub-cortical areas (striatum) and are responsible for a wide range of motor and cognitive functions that includes emotion regulation, movement, and attention (Beste et al., 2012). Dopamine plays a vital role in maintaining the normal function in the cortico-subcortical system (Carlsson and Carlsson, 1990; Cachope and Cheer, 2014; Kaiser et al., 2018) over the lifespan (Klostermann et al., 2012; Parr et al., 2021). Earlier animal studies have shown evidence of frontal cortex control over striatal dopamine release (Murase et al., 1993; Karreman and Moghaddam, 2002). Furthermore, animal and human experiments demonstrated that transcranial brain stimulation is able to induce significant release of dopamine and measurable changes in dopaminergic function in cortico-striatal networks (Bean et al., 1989; Strafella et al., 2001; Keck et al., 2002; Kanno et al., 2004). However, up to date little is known about the dose dependent effects of frontal stimulation on the striatal dopamine release in humans.

Neuroimaging techniques such as positron emission tomography (PET) provide the opportunity to quantify dopaminergic activity in the human brain (Badgaiyan, 2010; Kaiser et al., 2018). Neuroimaging studies employed multimodal combination of PET and non-invasive, repetitive transcranial magnetic stimulation (rTMS) (Paus et al., 1997, 2001; Chouinard et al., 2003; Ko et al., 2008; Cho and Strafella, 2009; Hallett et al., 2017). In their seminal study, Strafella et al. (2001) provided evidence of cortico-striatal control of dopamine release in the human brain, by applying rTMS on the left dorsolateral prefrontal cortex (DLPFC) and measuring the dopamine release in striatum using 11C-Raclopride. Their results displayed a significant dopamine release in the ipsilateral caudate. Later, they targeted the primary motor cortex with the same rTMS protocol and found evidence for induction of dopamine release in the ipsilateral putamen (Strafella, 2003).

Abnormalities in the fronto-striatal dopaminergic system are observed in movement disorders, such as Parkinson’s disease (Kish et al., 1988; Zgaljardic et al., 2003; Cropley et al., 2008), in schizophrenia (Grace, 1991; Lotze et al., 2009; Haber and Knutson, 2010; Lin et al., 2018) and in depression (Willner, 1983; Paus and Barrett, 2004; Furman et al., 2011; Li et al., 2015). Non-invasive brain stimulation is a potential tool for the treatment of neuropsychiatric disorders (Tremblay et al., 2020; Fried et al., 2021; Rossi et al., 2021). Therapeutical effects of rTMS are based on its capability to modulate brain activity and neurotransmitter release (Philip et al., 2019; Cole et al., 2020; Cristancho et al., 2020; Ishida et al., 2020; Zrenner et al., 2020). The DLPFC is one of the most frequently stimulated regions for therapeutic purposes, while it is highly interconnected with cortical and sub-cortical areas (Sibon et al., 2007; Cho and Strafella, 2009; Tik et al., 2017; Dowdle et al., 2018; Masina et al., 2019). Studies have shown, that the left DLPFC and right DLPFC possess an imbalance in activity, therefore providing high freuqency TMS on the left DLPFC accelerates the lower acitivity in the region (Johnson et al., 2013; Janicak and Dokucu, 2015). Considering the literature on the role of the DLPFC in depression, different DLPFC-rTMS protocols have been explored regarding their therapeutic potentials to improve depression symptoms (Bulteau et al., 2019; Mendlowitz et al., 2019; Ishida et al., 2020). Among them, the intermittent theta burst represents a reliable approach for its relatively short duration of application and its positive effects on adults with treatment-resistant depression (Li et al., 2014; Duprat et al., 2016). Indeed, iTBS can be delivered within 3 min (instead of the 37 min needed for a conventional 10 Hz rTMS treatment session) and demonstrated clinical effectiveness and safety at the same time (Huang et al., 2005; Blumberger et al., 2018; McGirr et al., 2021).

Stimulation intensity plays an important part in the effectiveness of iTBS protocols. By employing resting state functional Magnetic Resonance Imaging (rsfMRI) and different intensities of iTBS to the left DLPFC, our group demonstrated the threshold dependent modulation of fronto-striatal functional connectivity (Alkhasli et al., 2019). In particular, we applied iTBS at sub-threshold (90% rMT) and supra-threshold (120% rMT) intensities. Interestingly, the sub-threshold intensity was associated with a more reliable increase in functional connectivity between the DLPFC and bilateral Caudate Nucleus, as compared to supra-threshold intensity.

One important aspect of the present study is the choice of 18F-Desmethoxyfallypride (DMFP) which is a selective dopamine D2/D3 receptor radioligand (Mukherjee et al., 1996). The sensitivity of the radiotracer towards competition with dopamine allows the detection of changes in the levels of endogenous dopamine after the intervention of TMS. The binding of such a radiotracer has been shown to be inversely proportional to levels of dopamine concentration (Endres et al., 1997; Laruelle, 1997). This radioligand fulfils the demand of pharmacologic challenging studies because of its longer physical half-life of 110 min and its availability also at sites without a local cyclotron unit (Mukherjee et al., 2002; Gründer et al., 2003). During such long DMFP-PET measurements repeated iTBS became possible. Indeed, this is a crucial advantage over the 11C-Raclopride, that allows for shorter measurements given its much shorter half-life of 20 min (Schreckenberger et al., 2004; Li et al., 2014; Nettekoven et al., 2014; Hanlon et al., 2017). For example, Strafella in their two seminal studies (Strafella et al., 2001; Strafella, 2003) performed the TMS before the start of the 11C-Raclopride PET measurements.

In sum, the aim of the present study was to test the dose dependent effects of repeated bouts of iTBS over the left DLPFC on the dopamine release in the striatum. Therefore the analysis were restricted within the brain mask of the striatum. The repeated iTBS was delivered in intervals of 30 min. Stimulations inside the PET scanner were performed using neuro-navigation. We used DMFP as tracer, which allowed us to perform measurements lasting 130 min.

## 2 Materials and methods

### 2.1 Participants

A total number of 26 healthy participants were recruited; three of them were excluded from the analyses because they did not conclude their scans: one participant complained of back pain while laying in the PET scanner, two participants completed the first session but did not show-up for the second session. Analyses were conducted on data from 23 participants (nine females and fourteen males) with an age range of 18–65 years (mean age = 27.82, SD = 12.08), mean height of 174.60 cm (SD = 8.64) and mean weight of 74.78 kg (SD = 11.69). All of them were right-handed having a mean score of 95.77 (SD = 0.21) (quantified with the Edinburgh Handedness Inventory, Oldfield, 1971) and had no record of psychiatric or neurological disorder. Exclusion criteria were: contraindications for MRI (metals in the body such as magnetic dental implants, implantable neurostimulation systems, cathetors with metallic components, stents, piercings. etc), TMS (metal implants in the head such as cochlear implants, Implanted Cardioverter Defibrillator, Deep brain stimulator) and/or PET imaging: Claustrophobia; pregnancy, a test was performed on the day of each scan. All participants gave written informed consent after receiving full information on the study. They received a compensation of 350 € for participating in the study.

The study was in accordance with the Declaration of Helsinki and approved by the ethic committee (Protocol number: 003/15) of the Aachen University Hospital, RWTH Aachen University (Germany). Furthermore, the PET protocol was approved by the German authority for radiation protection in humans [Bundesamt für Strahlenschutz (BfS)].

### 2.2 Experimental procedure

In order to get the subjects familiar with the TMS protocol and to check their tolerability of the procedure a pre-screening session for each volunteer was performed. This included the application of the excitatory iTBS protocol to the prefrontal cortex with stimulation intensity equal to 90% of the individual resting motor threshold (rMT; procedure described below).

The experiment was conducted on three separate days for each participant. On day one, the screening for exclusion criteria, the informed written consent, and an MRI scan of the brain anatomy was acquired.

The verum and the sham stimulation sessions were administered on day two and day three, with their order counterbalanced between the participants.

### 2.3 Transcranial magnetic stimulation

The anatomical scan was integrated with a 3D model of the participant’s head obtained through an infrared neuro-navigation system (TMS navigator, Localite GmbH, Sankt Augustin, Germany). For the TMS equipment, a figure-of-eight coil (model: C-B60) was connected to a X100 MagPro simulator (MagVenture, Farum, Denmark). The Hotspot (M1 hand area) was determined visually by identifying anatomical landmarks in the primary morter cortex of each participant before delivering biphasic single pulses. The individual rMT was obtained through the collection of at least 5 motor evoked potentials (MEPs) over 10 stimulations with a peak greater than 50 uV. The stimulation intensity at the TMS machine was first increased in 2% steps until the determination of hotspot (M1 hand area), and then reduced stepwise to find out the lowest TMS intensity still inducing the supra threshold MEPs (greater than 50 uV). MEPs were recorded from the contralateral first dorsal interosseus (FDI) muscle of the right hand with pre-gelled surface electrodes. This procedure enabled us to determine the stimulation strength from M1 hand area, which is an important parameter and also a prerequisite for the stimulation at the brain target area.

The exact positioning of the TMS coil on the target site (iTBS over the DLPFC) of participant’s head was guided in real-time by the same previously described infrared neuro-navigational system. From behind the PET scanner, the TMS coil was fixed on the participant’s head with the help of a mechanical arm, whereas infrared cameras were placed in front of the PET scanner. Individual anatomical images were transformed into the Talairach system using the neuronavigation system (Localite TMS navigator), with the stimulation target (i.e., Dorsolateral Prefrontal Cortex) identified by the following Talairach coordinates (x, y, z): −45,45,35 (Fitzgerald et al., 2009). For the PET image analysis in SPM the MNI system was used.

**Figure 1:**
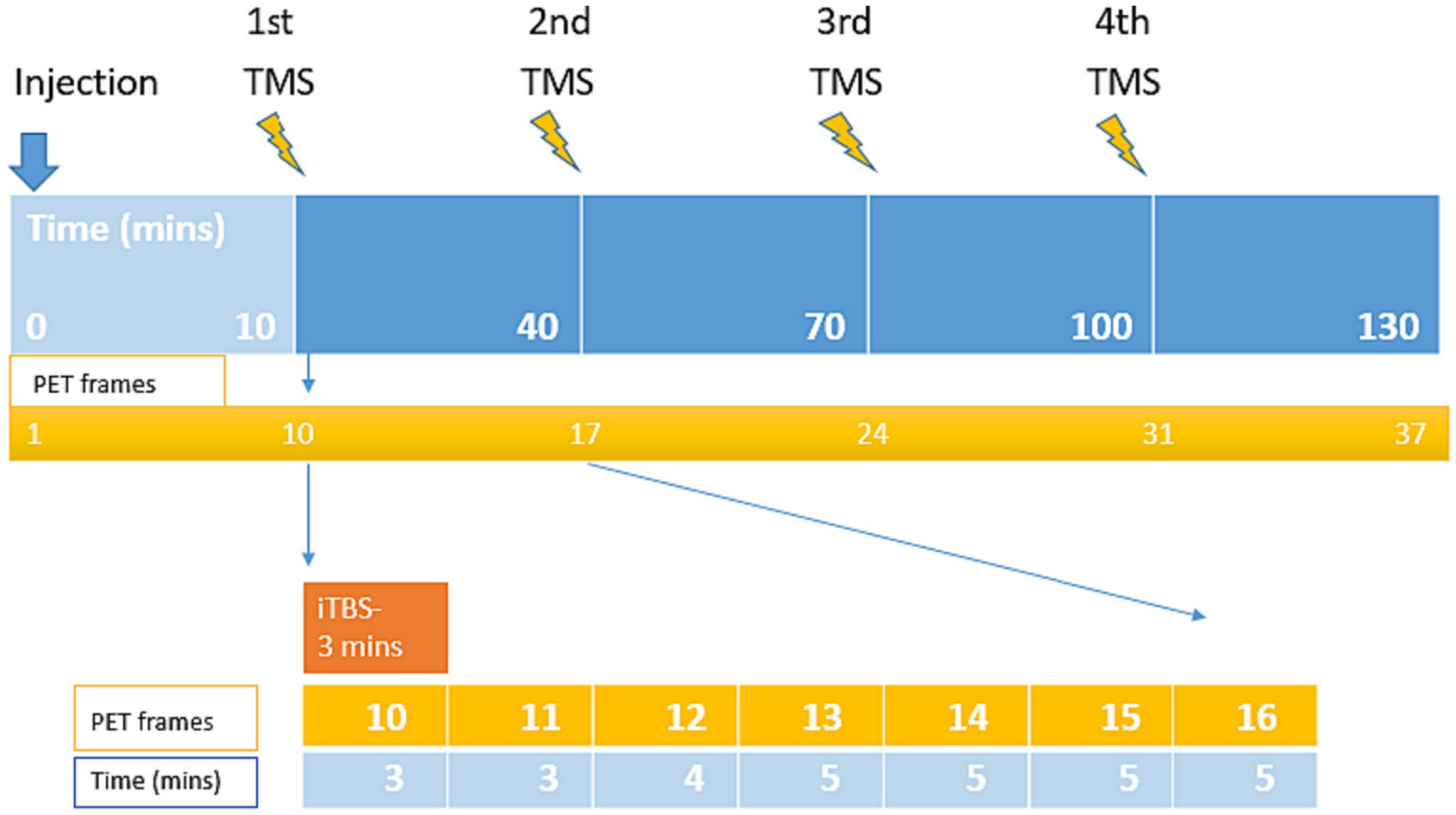
Visualization of the experimental design and durations of the combined TMS and PET measurement. The PET scan lasted 130 min, consisting of 4 iTBS, each delivered in 3 min stimulations delivered to the left-DLPFC at 30 min interval.

The sham and verum stimulation session, consisted of 4 excitatory iTBS (Huang et al., 2005) delivered to the left-DLPFC at 30 min interval (Figure 1). Excitatory iTBS protocol was comprised of 600 pulses, delivered in a sequence of 20 trains and 10 theta-bursts in a total duration of 190 s. Each 2 s long train consisted of a burst of 3 stimuli at 50 Hz, repeated in 5 Hz frequency and having inter-train interval of 8 s. In the verum condition, the stimulation intensities were set to 90% of the individual rMT (mean score = 38.65 ± 9.33). In the sham condition, the stimulation intensities were set at 30%, and the placebo coil was placed at the same target site as verum.

### 2.4 Magnetic resonance imaging

MRI scans were performed on a Magnetom Prisma 3.0 Tesla scanner (Siemens Medical Solutions, Erlangen, Germany). Anatomical data were acquired using a 3D magnetization-prepared, rapid acquisition gradient echo sequence (MPRAGE) with the following parameter: TR = 2,300 ms, TE = 2.98 ms, FOV = 256 mm, 176 sagital slices, slice thickness = 1 mm, in-plane resolution = 1 × 1 × 1 mm and matrix size of 256 × 256 × 128. The obtained T1 anatomical image from the MRI scanner was utilized for the imaging analysis including the setup for neuro navigation system and also for PET co-registration.

### 2.5 Positron emision tomography

The images were acquired on a Siemens ECAT EXACT HR+ scanner (Siemens-CTI, Knoxville, TN, USA). During the PET measurement, a 1-cm thick lead neck shield was used to limit scatter radiation arising from outside the field of view. PET data acquisition was initiated simultaneously with the bolus infusion of the radioligand (followed by a saline flush). A 18F-DMFP dose of approx. 200 MBq was administered to each of the participants per session. The doses (mean ± SD) were: 198.38 ± 7.66 at Day 1, and 201.42 ± 7.53 at Day 2 (total: 389.96 ± 10.02). PET data acquisition protocol comprised of a series of 37 time frames with a progressively increasing duration [3 × 20 s, 3 × 60 s, 3 × 120 s (2 min), 8 × 180 s (3 min), 4 × 240 s (4 min) and 16 × 300 s (5 min)].

PET scan was initiated simultaneously with the radiotracer injection. The first iTBS was delivered 10 min after the injection (i.e., into the uptake time of the radiotracer). In total, PET scan included 37 frames: the initial 9 frames (frame 1–9, total 10 min) were reserved for radiotracer uptake, and the remaining 28 frames (frame 10–37, total 120 min) were allocated for the experiment. For each block of Short iTBS (3 min), 7 gradually increasing PET frames were recorded.

18F-DMFP demonstrates as a highly reliable tracer for PET imaging of D2, D3 striatal dopamine receptors, which also acts as an efficient substitute for 11C-Raclopride, with the major advantage of carrying 18F-label, allowing the possibility for the transportation to PET facilitities (Mukherjee et al., 1996; Gründer et al., 2003). It also adds the benefit of using non-invasive reference methods without arterial blood sampling providing valid receptor quantities in human striatum region (procedure described below). 18F-DMFP shares the same intermediate affinity as 11C-Raclopride, but has a slight advantage for the binding in the extrastriatal regions like cortices (Mukherjee et al., 1996).

The dynamic molecular imaging technique used in this study detects the competition between the injected radiotracer and the endogenous dopamine for the occupancy of the same receptor binding sites. This competition results in the displacement of the radioligand from the receptor sites by the endogenous dopamine released by the TMS. Therefore, lower receptor bindings indicates the result of a higher dopamine concentration in the synaptic cleft (Badgaiyan et al., 2009; Badgaiyan, 2010).

### 2.6 Image pre-processing

The individual emission datasets were reconstructed per time frame by three-dimensional filtered back projection (Hamming filter, cut-off at 4 mm) algorithm resulting in 63 slices (2.425 mm thickness) using a128 × 128 image matrix (pixel size 2×2 mm). Datasets were fully corrected for photon attenuation, random coincidences, scatter radiation, and dead time. All image pre-processing procedures were performed using a dedicated software package (PMOD, version 3.8, PMOD Technologies, Zurich, Switzerland).

For each subject, the dynamic PET images were first automatically (mutual information algorithm) realigned to correct for potential effects of head movement. All PET processing were then performed according to an automatic protocol using the PMOD Fusion Tool (PFUS). Re-aligned PET images were first rigidly co-registered to individual Anatomical MRI scan. Then the individual MR images were spatially normalized and nonlinearly co-registered to the MNI space and the resulting transformation parameters were subsequently applied to each PET frame. All normalized co-registered images were visually checked for accuracy, and if necessary, manually adjusted.

For the region-based group comparison analysis of sham-verum stimulations, predefined masks were generated for each subject according to the WFU pick ATLAS. The predefined masks included the region of interest for caudate nucleus, putamen and cerebellum. Volume of Interests drawn on the normalized images were used to extract dynamic Time Activity Curves from the striatum and cerebellum region.

The standard methods including simplified reference tissue approach for the calculation of the binding of the radiotracer has an important prerequisite, stating that the condition of the system to be investigated is not amended. The application of the online-rTMS during the acquisition disturbs the system and creates an unstable condition. This violates the case and the valid outcome parameters such as Binding Potential and Relative Distribution Volume are not applicable here.

In order to describe the receptor behaviour in our TMS study, simple ratio method is implemented as an alternative approach which extracts the indices pointing to the receptor binding.

Receptor Binding Ratios were calculated from the simple ratio of specifically bound radioactivity in a receptor-rich (RC) and a receptor-free (RF) region, (RC/RF). The cerebellum (including the cerebellar hemispheres without the vermis) was used as the reference region, because of its lack of D2/3 receptors as described earlier (Siessmeier et al., 2005). Hence, simple ratios of striatal and cerebellar activities [S/C(t)] at different time points were derived for both sham and verum conditions, providing instantaneous values with changing over time.

Receptor Binding Ratios of the radiolabelled receptor ligands is inversely proportional to the concentration of dopamine. Therefore a reduction in the Receptor Binding Ratios in the striatum region after the TMS to the DLPFC suggest’s an increase in the amount of striatal dopamine.

As an alternative approach to the simplified reference tissue model, PET images were normalized to the cerebellum by dividing them with there corresponding cerebellar acitivties. Difference between the PET images (sham and verum condition) were calculated for the different time points. The resulting outcome is interpreted as indices of the neuroreceptor behaviour as a function of the rTMS.

For the voxel-based analysis on the images of binding ratios at each time point, paired t-test model was implemented in SPM 12 (Welcome Department of Imaging Neuroscience, London). The images were masked with a small search region (striatum). Direct contrast analysis (difference) between TMS condition (Sham vs. Verum) was calculated. *A-priori* striatal areas were defined using the masks from WFU_Pick-Atlas (SPM extension toolbox).

### 2.7 Statistical analysis

A within-participants repeated-measure analysis of variance (ANOVA) was performed on mean Receptor Bindings with *Area* (Caudate Nucleus vs. Putamen), *Hemisphere* (left vs. right), *Time Frame* (frame 10 to 37), and *TMS* (sham vs. verum) as within-subjects factors. A probability value of *p* = 0.05 was set as the significance threshold. Partial eta-squared (η^2^_p_) was calculated within the ANOVA as a measure of effect size.

All statistical tests were performed in SPSS (IBM, USA). When necessary, paired samples *t*-tests were performed as post-hoc comparisons with Bonferroni-corrected *p* value, as well as explorative difference (reverse Helmert) contrasts. An open-source tool was used to compute Cohen’s *d*_z_ effect size for the *t*-tests.^1^

## 3 Results

### 3.1 Analysis of variance

**Figure 2:**
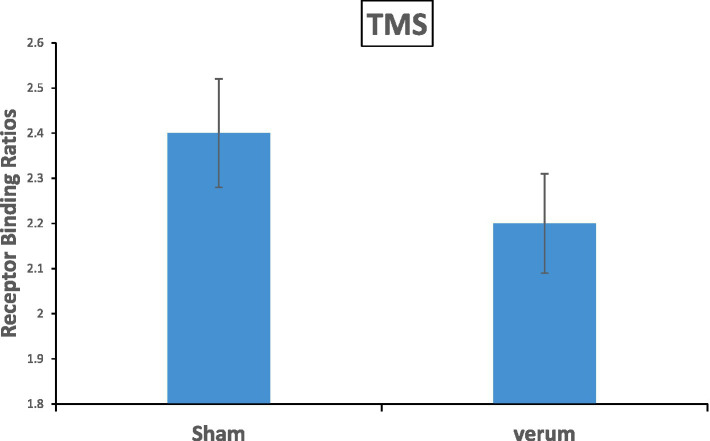
The main effect of TMS results in significance with a value of *p* = 0.02 on the Receptor Binding in the striatum. The verum stimulation showed a reduction of 8.6% 18F-DMFP Receptor Binding as compared to the sham stimulation. Error bars represents the standard errors.

Crucial for our hypothesis, the main effect of *TMS* resulted significant [*F*(1, 22) = 6.282, *p* = 0.020, η^2^_p_ = 0.22]: the Receptor Binding was reduced in the verum stimulation (mean Receptor Binding = 2.21) relative to the sham stimulation condition (mean Receptor Binding = 2.45) (Figure 2) and Table 1.

**Table 1:** Statistically significant repeated measures of ANOVA results.

Measure	Effect	SS	*df*	MD	*F*	*p*	η^2^_p_
TMS	Main effect	70.16	(1,22)	70.16	6.28	0.020	0.22
Time Frame	Main effect	783.01	(27,594)	29.00	239.12	0.000	0.22
TMS, Time Frame	Interaction	7.09	(27,594)	0.26	2.73	0.000	0.11
Area	Main effect	993.88	(1,22)	993.88	181.32	0.000	0.89
Area, Hemisphere	Interaction	25.36	(1,22)	25.36	16.58	0.000	0.43
Time Frame, Area	Interaction	33.88	(27,594)	1.25	18.82	0.000	0.46
Time Frame, Area Hemisphere	Interaction	4.24	(27,594)	0.15	7.134	0.000	0.24

Repeated measures analysis of variance results in significant main effects and interaction effects. Significance levels were computed using value of *p* < 0.05. SS, sum of squares; df, degrees of freedom; MS, mean squares; MD, mean difference; F, *F*-ratio; *p*, value of *p*; n^2^p, partial eta square.

**Figure 3:**
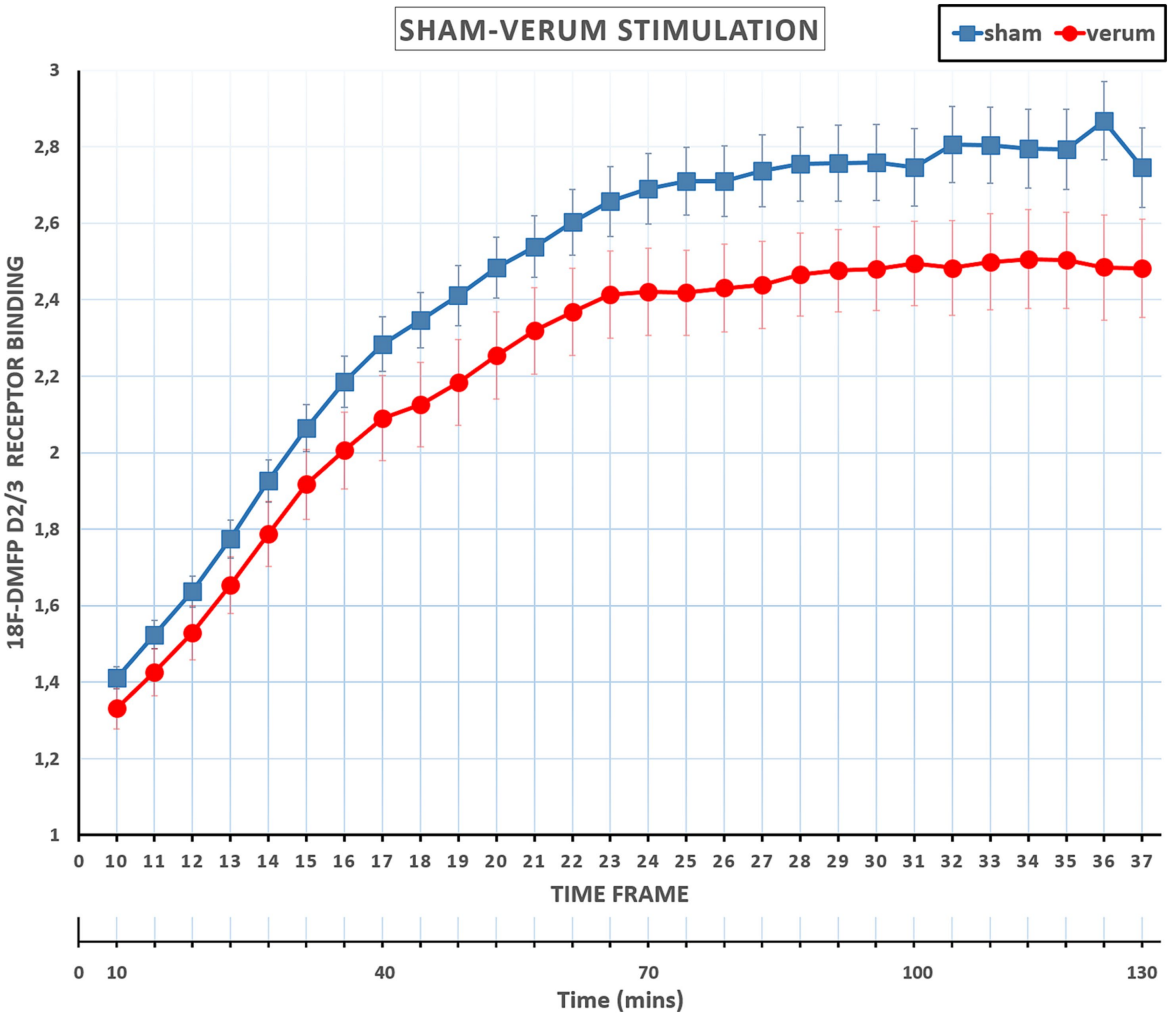
Mean Receptor Binding ratios at sham and verum TMSs across time frames 10 to 37. Receptor Binding ratios in the verum condition showed a progressive decrease from time frame 10 to time frame 28 (about 85 min) and then essentially stabilized until the end of the session, frame 37. Error bars represents the standard errors. PET scan included 37 frames (Total scan time of 130 min): the initial 9 frames (frame 1–9, total 10 min) were reserved for radiotracer uptake, and the remaining 28 frames (frame 10–37, total 120 min) were allocated for the experiment and also utilized for the interventions.

The main effect of *Time Frame* was significant [*F*(27, 594) =239.124, *p* < 0.001, η^2^_p_ = 0.92], displaying an increasing pattern of receptor binding over the time frame sequence (i.e., from frame 10 to 37). Importantly, the interaction between TMS and Time Frame was also significant [*F*(27, 594) = 2.731, *p* < 0.001, η^2^_p_ = 0.11] (see Figure 3). Difference contrasts (reverse Helmert contrasts) were applied to explore changes of the effect size (i.e., the receptor binding difference between sham and verum conditions) across the time frames. The effect size increased significantly at time frame 11 relative to time frame 10 [*F*(1, 22) = 5.210, *p* = 0.032, η^2^_p_ = 0.19], at time frame 14 [*F*(1, 22) = 5.107, *p* = 0.034, η^2^_p_ = 0.19], at time frame 16–20 [*F*s(1, 22) < =7.229, *p* < = 0.044, η^2^_p_ < = 0.25], 24 and 25 [*F*s(1, 22) < = 7.062, *p* < = 0.043, η^2^_p_ < = 0.24], and 27 and 28 [*F*s(1, 22) < = 5.937, *p* < = 0.031, η^2^_p_ < = 0.21], relative to the mean of previous frames. In other terms, compared to the sham condition, receptor bindings in the verum condition showed a progressive decrease from time frame 10 to time frame 28 and then stabilized until frame 37. No other interactions involved TMS.

ANOVA also revealed a significant main effect of *Area* [*F*(1, 22) = 181.323, *p* < 0.001, η^2^_p_ = 0.89], indicating that the Receptor Binding in the Putamen (mean Receptor Binding = 2.77) was higher than in the Caudate Nucleus (mean Receptor Binding = 1.89). The main effect of *Hemisphere* was not significant [*F*(1, 22) = 0.316, *p* = 0.584, η^2^_p_ = 0.01]. The interaction between Area and Hemisphere [*F*(1, 22) = 16.582, *p* < 0.001, η^2^_p_ = 0.43] was significant: Receptor Bindings were lower in the left Putamen (mean Receptor Binding = 2.72) relative to the right Putamen (mean Receptor Binding = 2.82) [*t*(22) = 3.261, *p* = 0.004, d_z_ = 0.67]. No difference was observed between the left Caudate Nucleus (mean Receptor Binding = 1.91) and the right Caudate Nucleus (mean Receptor Binding = 1.80) [*t*(22) = 1.617, *p* = 0.120, d_z_ = 0.34] (Bonferroni-corrected alpha level = 0.025).

Among other interactions, Time Frame interacted with Area [*F*(27, 594) = 18.828, *p* < 0.001, η^2^_p_ = 0.46] and with Area and Hemisphere [*F*(27, 594) = 7.134, *p* < 0.001, η^2^_p_ = 0.24]. These interactions are not crucial to test our experimental hypothesis, since they pooled together data from real (verum) and “fake” (sham) TMS. *Post-hoc*/pairwise comparisons are reported from these interactions for the sake of completeness. In the Area and Time Frame interaction, Putamen compared with Time Frames 10 to 27 results in significance [*t*(22) < = 18.170, *p* < = 0.016] and Time Frames 28 to 37 non-significance [*t*(22) < = 2.062, *p* > = 0.05], Caudate Nucleus compared with Time Frame 10 to 21 [*t*(22) < = 17.701, *p* < = 0.001] and Time Frames 22 to 37 non-significance [*t*(22) < = 1.571, *p* > = 0.05]. In the Interaction from Area, Hemisphere and Time frame, Putamen with left Hemisphere from Time Frame 10 to 21 shows significance [*t*(22) < = 22.012, *p* < = 0.001] and the Time Frames 22 to 37 non-significance [*t*(22) < = 8.011, *p* >= 0.05], Putamen with right Hemisphere Time Frames 10 to 21 shows significance [*t*(22) < = 22.024, *p* < = 0.001] and the Time Frames 22 to 37 non-significance [*t*(22) < = 9.043, *p* >= 0.05], also Caudate Nucleus with left Hemisphere from Time Frame 10 to 21 shows significance [*t*(22) < = 9.011, *p* < = 0.001] and the Time Frames 22 to 37 non-significance [*t*(22) < = 7.002, *p* > = 0.05], Caudate Nucleus with right Hemisphere Time Frames 10 to 28 shows significance [*t*(22) < = 9.023, *p* < = 0.001] and the Time Frames 29 to 37 non-significance [*t*(22) < = 7.033, *p* > = 0.05].

### 3.2 SPM analyses

**Figure 4:**
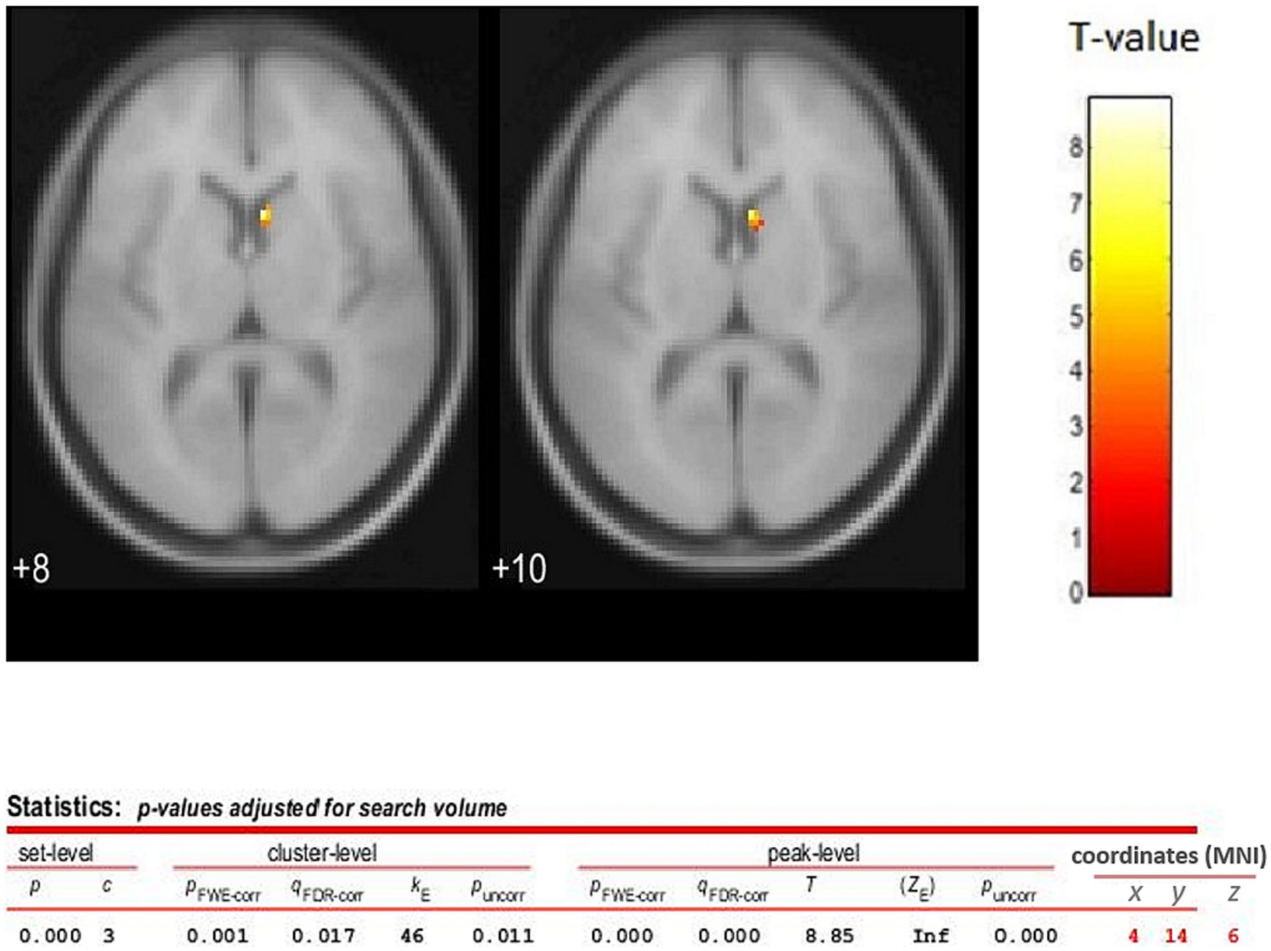
TMS sham vs. verum contrast (difference between the images) analysis for time frames 17 to 37 (2nd, 3rd, and 4th TMS blocks). The statistical parametric map (axial section) of the change in 18F-DMFP Receptor Binding is overlaid on the MNI stereotaxic space. The color scale represents the *T*-statistic. The parameters for displaying the image are as following, threshold *T* = 3.926 and value of *p* <0.05 (FWE). Release of dopamine is assessed using PET following four bouts of iTBS to the left DLPF cortex. Release is demonstrated by a reduction in 18F-DMFP binding. The results showed a separate cluster in the striatum region, the largest one having its peak at the coordiantes of *x* = 4, *y* = 14, *z* = 6 (Caudate Nucleus), and a size of 46 voxels. This observation shows that the prefrontal-striatal fibers can modulate the release of dopamine.

Analyses were restricted to data from time frames 17–37, that is after the second, third and fourth iTBS, where the Receptor Binding effect size was maximum and essentially stabilized across the time frames. The results showed a contralateral dopamine release in the basal ganglia region with one separate cluster, the largest one having its peak at the *x* = 4, *y* = 14, *z* = 6 coordinates (Nucleus Caudate). The peak *t* was 8.85 and the cluster size was 46 voxels (see Figure 4).

## 4 Discussion

The primary goal of the present study was to evaluate the dose dependent effects of iTBS to the L-DLPFC on dopamine release in the striatum using more advanced radiotracer and stimulation protocol than in earlier studies (Strafella et al., 2001). We wanted to disentangle the iTBS aftereffects on fronto-striatal connectivity by implementing neuro-navigated repeated bouts of iTBS, with 30 min interval, in a sham-controlled study. On the basis of the results we also wanted to find implications for the planning of therapeutic interventions with iTBS on L-DLPFC.

In line with our hypotheses, we found that the application of repetitive blocks of iTBS over L-DLPFC (verum stimulation) resulted in a significant increase of dopaminergic levels in the striatum (as reflected by decreased Receptor Bindings) compared to sham stimulation (Figure 2). Specifically, increased releases of dopamine were localized in the contralateral caudate nucleus (Figure 4). Importantly, dopamine levels in the verum condition increased progressively across the time frames until time frame 28 (approximately 85 min after the start of the session and after three iTBS bouts), and then essentially stabilized until the end of the session. In summary, our data show that repeated blocks of iTBS resulted in dose-dependent effects on the dopaminergic level with the enhancement of dopaminergic fronto-striatal connectivity. These results are in accordance with a previous study by Nettekoven et al. (2014), in which iTBS dose-dependent enhancement of brain connectivity was observed after stimulations of the Primary Motor Cortex.

The physiological mechanism behind the iTBS induced connectivity is that excitatory cortico-striatal projections can promote dopamine release by a local effect of glutamate on adjacent nigrostriatal nerve terminals (Chéramy et al., 1986). Such an effect may be mediated by glutamate receptors in the striatum, perhaps acting on dopamine nerve terminals (Hanbauer et al., 1992). These various observations from human (Strafella, 2003), primate studies (Ohnishi et al., 2004) and also between healthy subjects and patient groups (Pogarell et al., 2006, 2007) strongly emphasize how underlying neurochemical changes and the functional state of neuronal circuits along with different stimulation parameters may influence rTMS effects on striatal dopamine release.

The most important factor for the success of our study was the use of the radiotracer 18F-DMFP, which has a different data acquisition protocol in comparison to the previously used 11C-Raclopride tracer. The best direct comparison of the two ligands was performed by Siessmeier and colleagues (Siessmeier et al., 2005). It was demonstrated that the 18F-DMFP had a longer scan time as compared to the 11C-Raclopride. The use of DMFP allowed for a single injection followed by a dynamic acquisition of the effects of the repeated iTBS. Also important for our study was, that this tracer is not dependent on an onsite production due to the long half-life.

The findings of this study have not only significant theoretical, but also clinical implications.

Our study further supports the modulatory effects of TMS on dopaminergic circuits (Badgaiyan, 2010). The observation of increased dopaminergic activity in the Striatum is consistent with previous structural and functional studies that have implicated these areas as a consequence of TMS (Strafella et al., 2001; Strafella, 2003; Ko et al., 2008).

In patients with major depression, Pogarell and others (Pogarell et al., 2006) showed a reduction of 123I-IBZM Binding Potential (9.6%) in the striatum after left prefrontal stimulation. Using 11C-Raclopride PET, Kim et al. (2008) investigated the therapeutic effect of rTMS on motor cortex in PD patients. On two consecutive days, two sessions of rTMS induced a significant decrease of Binding Potential in the contralateral Caudate Nucleus (12.1%, but not ipsilateral to the stimulated hemisphere), while providing significant clinical benefits, as measured by the motor section of the Unified Parkinson’s Disease Rating Scale (UPDRS III). In fact, studies into synaptic plasticity have not only been an important driving force in neuroscience research but they are also contributing to the understanding of brain activity responses related to stimuli and finding new solutions to treat diseases.

Recently, comparison studies came with some striking observations of higher iTBS efficiency with respect to conventional rTMS protocols (Paus and Barrett, 2004; Cristancho et al., 2020). In patients with treatment-resistant depression, positive effects of iTBS were non-inferior to those obtained with 10 Hz rTMS. To note, the duration of a iTBS protocol is significantly reduced to a few minutes, compared to a rTMS protocol which normally lasts not less than 30 min. Furthermore, from a practical-operational point of view, by use of shorter iTBS protocols, either the number of stimulations within one session can be increased or the number of patients treated per day can be kept significantly higher without compromising clinical effectiveness (Blumberger et al., 2018).

TMS has been shown to normalize abnormal functional connectivity of cortico-cortical circuits in depression. Interestingly, Avissar and his group (Avissar et al., 2017), applied daily and over a 5 weeks period 10 Hz excitatory TMS on the left DLPFC. This protocol established higher functional connectivity between the DLPFC and striatum, which predicted better treatment response. Our study has shown comparable results and supports the use of TMS in clinical trials. More reliably, our findings suggest that three blocks of stimulation (spaced by 30 min interval) within one session are practicable and have a dose dependent additive effect on the dopamine activity in the striatum. This may plausibly correspond to increased therapeutic effectiveness of single therapeutic sessions.

## 5 Limitations

In the clinical application, it would be relevant to explore the effects on Dopamine also in patients. For the cumulative effects, it cannot be stated for sure that the increasing effects during the time course is solely due to repeated stimulation, i.e., it cannot be excluded completely that one bout alone results in delayed increasing effects. To test this, separate sessions with one, two, three and four bouts would have been required.

Considering the large age population (18–65) in the study, the effects might not be equivalent for the younger and older subjects. Also, here we have a mixture of men and women, the neuromechanisms for the effects of non-invasive brain stimulation might depend on the biological sex, see Rudroff et al. (2020) for an example in tDCS and Inghilleri et al. (2004) for example with rTMS.

Further research, incorporating a sample size with only men or women, with different combinations of stimulation protocols (e.g., continuous vs. intermittent theta burst), should be carried out. Furthermore, studies comparing healthy subjects and patients with depression and parkinson’s disease could lead to further understanding of the frontal-striatal network, and its effectiveness using with TMS as a possible treatment.

## 6 Conclusion

The repeated bouts of iTBS over the prefrontal cortex induced a dose dependent increase of regional prefrontal excitation and led to a modulation of the fronto-striatal network. These results provide an important contribution to the understanding of the mechanisms of cortically controlled dopamine release in the striatum.

This study also proposes a novel experimental approach – repeated blocks of iTBS – that allows to detect changes in radioligand binding during the uptake phase. Mapping of increased dopamine release was demonstrated in contralateral striatum in healthy subjects. For what concerns potential therapeutic applications, it is worth of mentioning that a three times repetition of iTBS can increase the effectiveness of the intervention, whereas further repetitions seem not to provide any additional benefit.

## Data availability statement

The raw data supporting the conclusions of this article will be made available by the authors, without undue reservation.

## Ethics statement

The studies involving humans were approved by Ethical committee, faculty of medicine, RWTH Aachen University (protocol number: 003/15). The studies were conducted in accordance with the local legislation and institutional requirements. The participants provided their written informed consent to participate in this study.

## Author contributions

US: Writing – original draft, Writing – review & editing, Conceptualization, Formal analysis, Investigation, Methodology, Software, Visualization. AP: Formal analysis, Investigation, Methodology, Software, Validation, Writing – review & editing. AS: Resources, Software, Visualization, Writing – review & editing. AH: Resources, Writing – review & editing. OW: Resources, Software, Visualization, Writing – review & editing. HH: Methodology, Validation, Writing – review & editing. FM: Conceptualization, Funding acquisition, Methodology, Project administration, Supervision, Writing – review & editing. FB: Conceptualization, Funding acquisition, Methodology, Project administration, Supervision, Writing – review & editing.

## Footnotes

^1^ https://memory.psych.mun.ca/models/stats/effect_size.shtml
